# Hereditary Spastic Paraplegia and Future Therapeutic Directions: Beneficial Effects of Small Compounds Acting on Cellular Stress

**DOI:** 10.3389/fnins.2021.660714

**Published:** 2021-05-06

**Authors:** Sentiljana Gumeni, Chiara Vantaggiato, Monica Montopoli, Genny Orso

**Affiliations:** ^1^Department of Cell Biology and Biophysics, Faculty of Biology, National and Kapodistrian University of Athens, Athens, Greece; ^2^Laboratory of Molecular Biology, Scientific Institute IRCCS Eugenio Medea, Bosisio Parini, Italy; ^3^Department of Pharmaceutical and Pharmacological Sciences, University of Padova, Padua, Italy

**Keywords:** hereditary spastic paraplegia, rapamycin, N-acetyl cysteine, salubrinal, guanabenz, methylene blue, naringenin, cell stress

## Abstract

Hereditary spastic paraplegia (HSP) is a group of inherited neurodegenerative conditions that share a characteristic feature of degeneration of the longest axons within the corticospinal tract, which leads to progressive spasticity and weakness of the lower limbs. Mutations of over 70 genes produce defects in various biological pathways: axonal transport, lipid metabolism, endoplasmic reticulum (ER) shaping, mitochondrial function, and endosomal trafficking. HSPs suffer from an adequate therapeutic plan. Currently the treatments foreseen for patients affected by this pathology are physiotherapy, to maintain the outgoing tone, and muscle relaxant therapies for spasticity. Very few clinical studies have been conducted, and it’s urgent to implement preclinical animal studies devoted to pharmacological test and screening, to expand the rose of compounds potentially attractive for clinical trials. Small animal models, such as *Drosophila melanogaster* and zebrafish, have been generated, analyzed, and used as preclinical model for screening of compounds and their effects. In this work, we briefly described the role of HSP-linked proteins in the organization of ER endomembrane system and in the regulation of ER homeostasis and stress as a common pathological mechanism for these HSP forms. We then focused our attention on the pharmacodynamic and pharmacokinetic features of some recently identified molecules with antioxidant property, such as salubrinal, guanabenz, N-acetyl cysteine, methylene blue, rapamycin, and naringenin, and on their potential use in future clinical studies. Expanding the models and the pharmacological screening for HSP disease is necessary to give an opportunity to patients and clinicians to test new molecules.

## Introduction

Hereditary spastic paraplegia (HSP) is a genetically heterogeneous group of neurodegenerative diseases characterized by progressive spasticity and weakness at the lower limbs. Clinically, HSP has been classified as “pure” (or “uncomplicated”) and “complex” (or “complicated”) forms (Parodi et al., 2017). The main clinical features of pure HSP are hyperreflexia, hypertonicity or bilateral spasticity of the legs, bladder dysfunction, and vibratory sense impairment. Complicated forms show also additional neurological or extra-neurological symptoms such as intellectual disability, cerebellar ataxia, peripheral neuropathy, epilepsy, retinopathy, optic atrophy, dystonia, and Parkinsonism (Boutry et al., 2019a). Genetically, HSPs can be divided in autosomal dominant (AD), autosomal recessive (AR), and X-linked forms. To date, 79 loci have been mapped on different chromosomes corresponding to 68 genes (spastic gene or SPG). The common pathological feature of HSP is the retrograde axonal degeneration of the distal portions of corticospinal and spinocerebellar tracts, corresponding to the longest motor and sensor axons of the central nervous system (CNS). Although little is known about the mechanisms related to neurodegeneration in complicated forms, the functional studies carried out in recent years on HSP-linked genes have suggested that alterations of intracellular trafficking may be a common element. The affected cellular functions include lipid metabolism, active axonal transport, organelle shaping, and the endo-lysosomal system (Boutry et al., 2019a; Darios et al., 2020; Öztürk et al., 2020). Although the progress made in deciphering the pathological process underlying HSP, there is still no specific cure to prevent or slow down neuronal degeneration or dysfunction. Of note, some of the pathways deranged in HSP are common with other neurodegenerative diseases, making the therapy against neurological deterioration of the nervous system even more difficult. To date, the treatments for HSP are based on antispastic drugs, botulinum toxin, and physiotherapy (Bellofatto et al., 2019; Paparella et al., 2020), and clinical trials on HSP patients have generated only few positive results.

**FIGURE 1:**
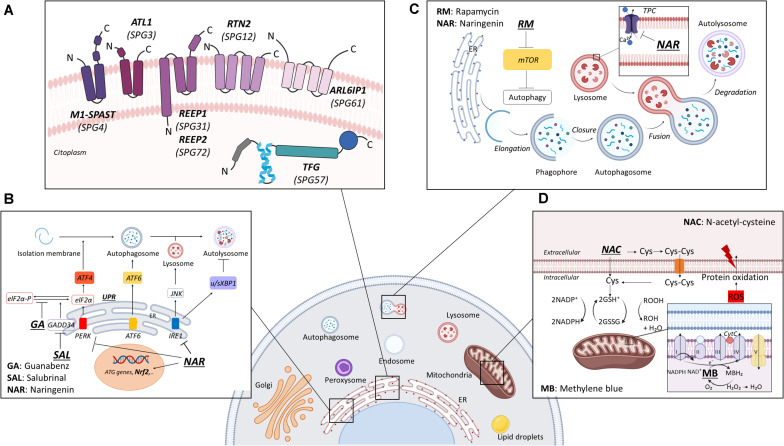
Summarized molecular targets of the small molecules as hereditary spastic paraplegia (HSP) therapeutics. **(A)** Schematic view of the ER-linked HSP proteins and their localization. **(B)** Guanabenz, salubrinal, and narigenin are involved in the reduction of ER stress, acting on UPR components or Nrf2, the master regulator of antioxidant responses. **(C)** Rapamycin specifically binds to mTOR and activates autophagy, whereas NAR inhibits TPC channels on lysosomes. **(D)** NAC is an aminothiol and synthetic precursor of intracellular cysteine and glutathione (GSH), acting as an antioxidant or free radical scavenger. Abbreviations: ER, endoplasmic reticulum; UPR, unfolded protein response; Nrf2, nuclear factor erythroid 2-related factor 2; PERK, protein kinase R-like ER kinase; ATF6, activating transcription factor 6; IRE1, inositol-requiring enzyme 1; eIF2α, eukaryotic initiation factor 2α; eIF2αP, phosphorylated eukaryotic initiation factor 2α; GADD34, growth arrest and DNA damage-inducible protein; mTOR, mammalian target of rapamycin complex 1; ROOH, organic hydroperoxide; cys, cysteine; Cys-Cys, cysteine; cyt, cytochrome c; ROS, reactive oxygen species; TPC, two-pore channel. Created with BioRender.com.

A new opportunity comes from a group of HSP-associated genes, all acting in a common pathway as modifiers/regulator of the endoplasmic reticulum (ER). Indeed, a fine connection between specific HSP-related genes and ER homeostasis and morphology has been identified. Atlastin (*SPG3A*), spastin (*SPG4*), receptor expression-enhancing protein 1 (REEP1) (*SPG31*), reticulon 2 (*SPG12*), TGF (*SPG57*), ARL6Ip1 (*SPG61*), and REEP2 (*SPG72*) are all involved in maintaining the ER morphology and in ER stress regulation, making it a common pathological phenotype of the HSP subtypes associated with these proteins (Figure 1). We discuss the role of these HSP-related proteins in ER homeostasis and how this can affect other intracellular processes. Microtubule-targeting compounds previously identified have shown beneficial effects in HSP models, but the high toxicity and adverse effects prevent their clinical repositioning for pathologies such as neurodegeneration (Orso et al., 2005; Wali et al., 2020). Here, we focus on new effective compounds tested in *in vivo* models, such as *Drosophila melanogaster*, *Danio rerio*, and *Caenorhabditis elegans*, which could be considered for HSP therapy. Furthermore, we analyze and describe the pharmacological property of the Food and Drug Administration (FDA)-approved molecules with antioxidant activities that can be considered in the therapy of these ER-related HSP subtypes.

## HSP Pathways

### HSP Genes as Modulators of ER Morphology and Stress

The ER is a single membrane-bound organelle involved in many cellular processes including protein synthesis and transport, protein folding, lipid synthesis, carbohydrate metabolism, calcium homeostasis, detoxification, storage, and action of enzymes, lipid droplet (LD) formation, and metabolism (English et al., 2009; Chen et al., 2013). ER includes a nuclear envelope, a system of flattened cisternae that separates the nucleus from the cytoplasm deputed to protein synthesis, and a peripheral tubular structure extending in to the cytoplasm connecting the cortical ER to the nuclear envelope (Shibata et al., 2006; West et al., 2011; Phillips and Voeltz, 2016; Wu et al., 2017). ER sheets are flat structures consisting of two lipid bilayers, covered with ribosomes, whereas ER tubules are cylindrical structures with high membrane curvature at their cross-section that are connected by three-way junctions. The relative abundance of sheets or tubules correlates to cell type/function (Puhka et al., 2012; Shemesh et al., 2014).

The structural organization of ER is created and maintained thanks to a continuous process of membrane remodeling governed by homotypic fusion events, tubulation and curvature rearrangements, as well as by cytoskeletal transport (Fowler et al., 2019; Öztürk et al., 2020). Some of the main players of this process are proteins involved in HSP. Mutations in REEP1 (receptor expression-enhancing protein 1), REEP2, and reticulon 2, which cause SPG31, SPG72, and SPG12, respectively, are some of the main ER-shaping proteins (Voeltz et al., 2006; Tolley et al., 2010; Zurek et al., 2011; Esteves et al., 2014). The loss of reticulon and REEP1 in *Drosophila* and the overexpression of REEP2 mutant form in human cells induce the expansion of ER sheets (O’Sullivan et al., 2012; Yalçın et al., 2017; Napoli et al., 2019). In addition, mutations in the PB1 domain of TFG (trafficking from ER to Golgi regulator), a highly conserved regulator of protein secretion that functions at the interface between the ER and ER–Golgi intermediate compartments, significantly reduce the self-assemble ability of the protein, while its loss leads also to ER expansion (Witte et al., 2011; Elsayed et al., 2016; Steinmetz et al., 2020). Atlastin1, a transmembrane protein with GTPase activity associated with SPG3A, drives the generation of ER three-way junctions (Hu et al., 2009; Orso et al., 2009; Pendin et al., 2011) and modifies the ER morphology by homotypic membrane fusion. In addition to these HSP proteins participating in membrane shaping, spastin, which is mutated in SPG4, has been found to disassemble and remodel neuronal microtubules and to maintain ER structure integrity and calcium homeostasis (Orso et al., 2005; Evans et al., 2006; Sanderson et al., 2006; Vajente et al., 2019).

The ER plays a crucial role in quality control of newly synthesized proteins through two interconnected pathways: the unfolded protein response (UPR) and the ER-associated protein degradation (ERAD). UPR activation increases the folding capacity of the ER, while the ERAD system promotes misfolded protein identification and their degradation in the cytoplasm by the ubiquitin–proteasome system. In this way, the ER maintains the flow of protein synthesis, folding, and clearance (Zhang et al., 2002, 2005; Wu et al., 2007; Yamamoto et al., 2007; Walter and Ron, 2011; Leitman et al., 2014; Sekiya et al., 2017; Hwang and Qi, 2018; Vitale et al., 2019).

ER stress is a condition activated by various stimuli including those of cellular redox regulation or by the accumulation of unfolded proteins in the ER, triggering the evolutionarily conserved pathway UPR (Gumeni et al., 2017). UPR is activated through three signaling cascades by the ER transmembrane sensor proteins PERK (protein kinase R-like ER kinase), IRE1 (inositol-requiring enzyme 1), and ATF6 (activating transcription factor 6) (Inagi et al., 2014). PERK suppresses the cellular protein synthesis and the protein flux into the ER by phosphorylating the α-subunit of the eukaryotic initiation factor 2 (eIF2α). The activation of its target, the activating transcription factor 4 (ATF4), resumes the translation or induces apoptosis if the ER stress persists, activating the apoptotic protein CHOP and the transcription factor GADD34. IRE1 activates the transcription factor XBP1 (X-box binding protein) that induces the expression of many UPR genes contributing to ER-associated degradation and counteracts ER stress activating the c-Jun N-terminal kinase (JNK) pathway and inducing JNK-mediated autophagy and apoptosis. ATF6 translocates to the Golgi apparatus where it is processed to create a highly active transcription factor that activates the UPR transcription factors GADD153 and XBP1 and ER chaperones to increase ER folding capacity (Almanza et al., 2019). The increase of protein synthesis requires the expansion of the ER membrane network, thus associating UPR with ER membrane extension and remodeling (Wiest et al., 1990; Mandl et al., 2013). It has been reported that ER membrane expansion and generation of new ER sheets could act as a stress-alleviating response independently of UPR activation, suggesting that ER expansion is an integral part of an effective UPR (Schuck et al., 2009). Moreover, modulation of UPR after the disruption of optimal membrane rearrangements has already been reported in *Drosophila*. The downregulation of the ER-shaping protein Rtln1 determines a partial loss of tubular ER and a significant increase of the ER stress response in epidermal cells and neurons (O’Sullivan et al., 2012); the expression of *RTN3*, a specific receptor involved in ER tubule degradation, is upregulated by ER stress and its loss attenuates basal ER stress (Chen et al., 2011; Grumati et al., 2017); the *Arabidopsis* mutants of the atlastin GTPase homologue *RHD3*, which have long unbranched ER tubular structures, lack the ability to invoke UPR by interfering with Ire1 function (Lai et al., 2014). In support, the loss of *ReepA* (*REEP1* ortholog) in *Drosophila* triggers a selective activation of Ire1 and Atf6 and modifies ER morphology (Napoli et al., 2019). Also, TFG is likely associated with disruption of intracellular protein homeostasis and ER stress, since it inhibits the protein degradation system, resulting in an increase of ER resident and ER stress-related proteins (Yagi et al., 2014). Although the mechanism by which these ER-shaping proteins regulate UPR is still unclear, a link between tubular ER structure and ER stress is being uncovered.

### ER and Lipid Droplets Interplay in HSP Models

Lipid droplets (LDs) are cellular specialized organelles that store neutral lipids in all living organisms. They are composed by a core, containing mainly triacylglycerols (TAG) and sterol esters (SE), enclosed by a single phospholipid and protein layer (Olzmann and Carvalho, 2019; Thiam and Ikonen, 2021).

Unlike most other organelles, LDs are not formed by growth and fission of existing droplets, but they are likely formed *de novo* from the ER membranes (Jacquier et al., 2013). Lipids accumulate between the cytoplasmic leaflets of the ER membrane, where the two enzymes diacylglycerol acyltransferase 1 and 2 (DGAT1 and DGAT2) synthesize triglycerides. As the volume increases, the leaflet swells as a globular mass until it is pinched off from the membrane to become an independent LD (Suzuki et al., 2011).

In mammals, the LD synthesis is characterized by three main steps: neutral lipid synthesis, LD formation, and growth. *De novo* TAG synthesis occurs in a four-step pathway involving glycerol-3-phosphate O-acyltransferase (GPAT), 1-acylglycerol-3-phosphate O-acyltransferase (AGPAT), phosphatidic acid phosphatase (PAP) (or lipin), and DGAT enzymes. At the last step of the pathway, fatty acids, firstly activated to acyl-CoA, are converted to TAGs through DGAT1 and DGAT2 enzymes (Onal et al., 2017).

The importance of the LD in HSP mechanism is highlighted by recent evidences showing that the HSP-related proteins seipin/*SPG17*, Erlin2/*SPG18*, atlastin/*SPG3A*, spartin/*SPG20*, spastin/*SPG4*, and REEP1/*SPG31* localize on LD or affect LD turnover (Darios et al., 2020). Seipin, responsible for the Silver syndrome (*SPG17*), is a critical regulator of human adipose tissue development (Szymanski et al., 2007; Tian et al., 2011). Seipin localizes and regulates ER-LD contacts and the incorporation of proteins and lipids in LDs in human fibroblasts (Salo et al., 2016). Spartin interacts with the surface of LD lipid monolayer through its C-terminal region and plays a role in LD regulation by binding to TIP47 and E3 ubiquitin ligases, leading to degradation of LD-associated proteins (Eastman et al., 2009; Edwards et al., 2009; Hooper et al., 2010; Urbanczyk and Enz, 2011). The GTPase atlastin has been shown to regulate LD size in *C. elegans* and *D. melanogaster* and to induce the formation of larger LDs after co-expression with REEP1 in mammalian cells (Klemm et al., 2013). Moreover, mutated forms of human REEP1 are found to localize in LD in cell cultures (Falk et al., 2014), while *Reep1^–/–^* mice showed an impairment of LDs and lipoatrophy (Renvoisé et al., 2016). On the other hand, spastin regulates the contact between LD and peroxisomes, facilitating fatty acid trafficking (Chang et al., 2019), mediates the dispersion of LDs from the ER upon glucose starvation, in a microtubule-dependent manner, and preserves the morphogenesis of the ER when TAG synthesis is prevented (Arribat et al., 2020; Tadepalle et al., 2020).

LDs are particularly important in tissues specialized for energy storage or lipid turnover, such as the adipose tissue, the liver, and the intestine, and accumulate in skeletal muscles and nervous system (Missaglia et al., 2019). LDs not only provide substrates for energy metabolism and building blocks for membranes but also play a pivotal role in various cellular pathways, like protein trafficking, protein degradation, and modulation of nuclear receptors. Moreover, LDs exhibit a protective function against oxidative damages induced by different stimuli leading to ER stress (Fei et al., 2011; Zhang and Zhang, 2012; Wilfling et al., 2013; Chitraju et al., 2017). The disruption of triacylglycerol synthesis and LD biogenesis induce UPR activation in yeast (Velázquez et al., 2016; Olzmann and Carvalho, 2019) and mammalian cells (Chitraju et al., 2017), while ablation of the ER protein Rab18 in preadipocytes activates UPR upon oleate treatment (Xu et al., 2018).

### ER and Endolysosomal Connection in HSP

Material is internalized in the cells in clathrin-coated vesicles that originate from the plasma membrane and rapidly fuse with early endosomes, a sorting compartment from which molecules are recycled to the plasma membrane or transported to lysosomes for degradation (Zhang et al., 2011). Endocytic organelles are extremely dynamic, and many sorting, fusion, and fission events occur along the pathway. The establishment of dynamic contacts between the ER and the endolysosomal compartment is involved in the regulation of cargo sorting and endosome dynamics, including endosome/lysosome positioning, endosome fission, cholesterol transfer, calcium mobilization and signaling, lysosome biogenesis, and receptor dephosphorylation (Raiborg et al., 2015a,b; Eden, 2016). The first ER–endolysosomal contact site to be characterized is the interaction between the integral ER membrane protein VAPA/B (vesicle-associated membrane protein-associated A/B) and the lysosomal sterol binding proteins ORP1L (oxysterol-binding protein homologue), StARD3 (StAR-related lipid transfer domain containing 3), and StARD3NL (STARD3 N-terminal-like proteins). This contact is involved in the regulation of endosomal fusion and trafficking and in cholesterol transfer (Fowler et al., 2019). As much as 30% of lysosome-associated cholesterol is transferred to the ER, and VAP-ORP1L and VAP-StARD3 contact sites play a major role in this. Indeed, these contact sites favor the interaction between intracellular cholesterol transporter 1 (NPC1) on lysosomes and the integral ER membrane protein oxysterol-binding protein-related protein 5 (ORP5) that transfers the cholesterol from lysosomes to the ER (Van Der Kant and Neefjes, 2014).

ER–lysosome contact sites are also involved in the regulation of calcium flux between the two organelles (Kilpatrick et al., 2013). Lysosomal calcium efflux, mediated by the second messenger NAADP (nicotinic acid adenine dinucleotide phosphate), activates the inositol 1,4,5-trisphosphate (IP3) receptor on the ER, inducing the release of calcium from this organelle. Calcium efflux from the ER also regulates lysosomal calcium efflux. This reciprocal regulation is promoted by a close association between the IP3 receptor on the ER and LAMP1/Rab7 on lysosomes (Morgan et al., 2013). Lysosome functionality affect the formation of this contact site: inhibition of lysosomal acidification reduces IP3R–LAMP1 interaction inducing lysosome enlargement and altering the calcium flux.

Several proteins, such as protrudin and motor proteins, participate in the regulations of ER–LE contacts and in the endosome transport. Protrudin is an integral ER membrane protein that mediates ER–LE contacts interacting with VAPA on the ER and with Rab7 and phosphatidylinositol 3-phosphate (PI3P) on LE. These contacts are required for the transfer of kinesin heavy chain isoform 5A (KIF5A), a motor protein that mediates the anterograde vesicle transport, to LE. KIF5A interacts with protrudin on the ER and with the motor protein adaptor FYVE and coiled-coil domain-containing protein 1 (FYCO1) on late endosomes. After dissociation from protrudin, KIF5A binds to microtubules and, through the interaction with FYCO1, promotes the motility of LE to cell periphery and the formation of neurites (Raiborg et al., 2015a; Shirane, 2020). The protrudin–KIF5A complex plays a central role in the regulation of vesicular trafficking and alteration in these ER–LE contact sites and in motor proteins contributes to the pathogenesis of HSP. Mutations in KIF5A are associated with the HSP form SPG10 and induce mitochondrial and lysosomal transport defects. Moreover, protrudin, together with spastin, promotes microtubule motor-dependent movement of late endosomes toward the plasma membrane, with which they fuse, delivering membrane to drive protrusion formation (Connell et al., 2020). Protrudin colocalizes at the tubular ER with other ER proteins associated with HSP forms, spastin (*SPG4*), atlastin1 (*SPG3A*), and REEP1 (*SPG31*), and its overexpression also promotes the formation and stabilization of this network (Hashimoto et al., 2014).

ER–endosome contact sites define also the position and timing of endosomal fission that plays an important role in endosome maturation and recycling to the plasma membrane (Rowland et al., 2014). Recycling of receptors, such as the transferrin receptor (TfnR) or mannose 6-phosphate receptors (M6PRs), back to the plasma membrane or to the Golgi apparatus requires the formation of tubular protrusion on early or late endosomes. Cargoes destined for recycling back to the trans-Golgi network (TGN) accumulate in the narrow tubules, while those targeted for lysosomal degradation remain in the larger vacuolar portion (Lee and Blackstone, 2020). Cargo sorting into the endosomal protrusions involve the cargo-sorting retromer complex and the Wiskott–Aldrich syndrome protein and SCAR homologue (WASH) complex. After cargo sorting, the ER protein VAP interacts with FAM21-strumpellin, components of the actin–regulatory WASH complex, inducing the formation of ER–endosome contact sites and defining the site of membrane constriction. This process requests ER-shaping proteins, such as REEP1, to induce the membrane curvature that promotes endosomal constriction, and proteins, such as spastin, that promote microtubules severing (Allison et al., 2017). Both M1 spastin and its smaller isoform M87 interact with the endosomal sorting complexes required for transport III (ESCRT-III protein) Ist1, and this interaction is required for mediating ER–endosome contacts important for ER-induced endosomal tubule fission. Even in this case, the majority of the proteins involved in endosomal fission are HSP-related proteins. Mutations of spastin/SPG4 result in impaired endosomal fission, defects in M6PR sorting, defects in lysosomal enzyme trafficking, and abnormal lysosomal morphology (Connell et al., 2020). Moreover, mutations in the WASH complex component strumpellin/WASHC5 impair endosomal fission and are associated with the HSP form SPG8. Also, the dysregulated expression of the ER-shaping protein RTN4 inhibits endosome fusion (Rowland et al., 2014). The failure of ER-mediated endosomal fission causes abnormal lysosomal morphology. Indeed, strumpellin mutations result also in a reduction of lysosomal number and in lysosome enlargement (Allison et al., 2017; Song et al., 2018). Endosomal or lysosomal abnormalities with the accumulation of enlarged lysosomes have been also observed in primary neurons from a SPG4 mouse model and in SPG4 patient-derived fibroblasts (Allison et al., 2017; Rehbach et al., 2019) and in mouse neurons lacking REEP1 (Allison et al., 2017).

### ER Homeostasis and Autophagy in HSP

Autophagy and ER stress can modulate each other, since ER stress response can either activate or inhibit autophagy by regulating autophagy gene expression. Specifically, the activation of the PERK–eIF2α–ATF4 pathway upregulates the expression of a large set of autophagy genes, while IRE1 signaling has been implicated in either promoting autophagy via JNK-mediated signaling or in eliciting a negative regulation (Rashid et al., 2015; De Leonibus et al., 2019; Zheng et al., 2019). On the other hand, the autophagy-selective degradation of ER components, called ER-phagy, regulates ER homeostasis, thus emerging as an essential protective mechanism during ER stress (Rashid et al., 2015; De Leonibus et al., 2019; Zheng et al., 2019). ER-phagy is also involved in an additional quality control mechanism for misfolded ER proteins/components that are not eligible for ERAD (De Leonibus et al., 2019). The process, reported as ER-to-lysosome-associated degradation (ERLAD), involves the following: (1) ER-phagy (generation of autophagosomes and inclusion of ER membranes that fuse with lysosomes for degradation); (2) micro-ER-phagy (lysosomal membranes invaginate and remove parts of the ER into the lysosomal lumen); and (3) vesicular delivery (ER-derived vesicles fuse with lysosomes for degradation) (Chino and Mizushima, 2020). Therefore, a functional autophagosomal–lysosomal system and a correct lysosomal recycling are essential to ER quality control (De Leonibus et al., 2019).

Autophagy and lysosomal abnormality have already been described in several HSP forms, involving proteins that localize in endosomes, such as spatacsin, spastizin, and AP5 complex members (Vantaggiato et al., 2013, 2019; Renvoisé et al., 2014; Varga et al., 2015; Hirst et al., 2018; Khundadze et al., 2019). Spatacsin or spastizin mutations are associated with the HSP subtypes *SPG11* and *SPG15*, respectively, and result in lysosome depletion and accumulation of autophagosomes (Vantaggiato et al., 2013; Chang et al., 2014; Varga et al., 2015). In addition, mutations in AP-5 complex subunit zeta-1 (*AP5Z1)*, which are associated with SPG48, induce the accumulation of endolysosomes containing aberrant storage material (Hirst et al., 2015). A latest report also suggests that TFG controls autophagy flux in CH12 B lymphoma cells (Steinmetz et al., 2020).

Spatacsin and spastizin are both involved in ALR, a process of lysosome reformation from autolysosomes after cargo degradation, which recycles lysosomal components (Chang et al., 2014). During ALR, tubular structures extrude from the autolysosome to originate a proto-lysosome, which lacks autophagic components and matures acquiring acidity and degradative components. Spatacsin mutations inhibit tubule formation, inducing the accumulation of lipid, gangliosides, and cholesterol in the lysosomes and impairing cholesterol trafficking and calcium homeostasis (Boutry et al., 2018, 2019b). Indeed, SPG11-mutated cells present low cholesterol levels in the plasma membrane and an increase in the number of contacts between the ER and the plasma membrane, which are involved in lipid transfer and in the regulation of calcium homeostasis. This increase the import of extracellular calcium and the levels of cytosolic calcium. It could be interesting to analyze ER stress and homeostasis in these HSP forms associated with genes involved in autophagy regulation and lysosomal functions. This may be of relevance for the evaluation of the possible effects of autophagy and lysosomes modulating drugs in ER-related HSP forms.

## Therapeutic Strategies

Clinical treatment of HSPs consider the management of the spasticity by specific exercise programs or oral administration of antispasmodics, including gabapentin, tizanidine and baclofen (delivered also intrathecally via a baclofen pump), or botulinum. In addition, oxybutynin or tolterodine can treat bladder dysfunction (Trummer et al., 2018; Bellofatto et al., 2019; Shribman et al., 2019). However, apart from treating the spasticity of HSP cases, little attention is given to other symptoms.

**FIGURE 2:**
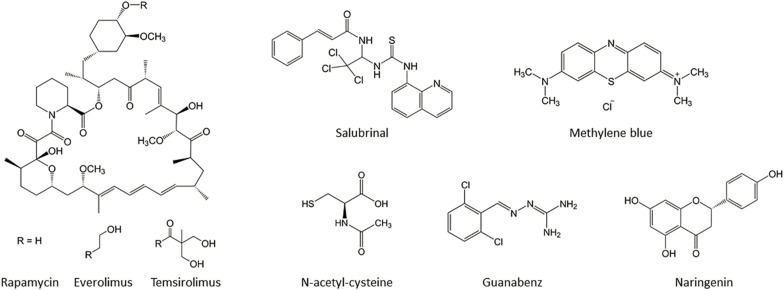
Chemical structure of the molecules that could be considered for the treatment of HSP disease.

Considering the extensive genetic heterogeneity, the consequent mechanistic diversity, and the different progression of clinical cases, to date there are no effective HSP therapy available. Therefore, researchers have additionally focused their attention on mechanistic approaches to the therapy. The following sections summarize potential therapeutic molecules with antioxidant and neuroprotective properties, such as salubrinal, guanabenz, N-acetyl cysteine, methylene blue, rapamycin, and naringenin (Figure 2), which are involved in UPR response, reactive oxygen species (ROS) production, and autophagy regulation (Figure 1), that could represent a therapeutic strategy for the HSP forms associated with altered ER homeostasis and ER stress.

### Approved FDA Drugs Used in Clinical Trials

This section is dedicated to the drugs that have been validated for a therapeutic use by the ruling authority of the FDA. The following drugs have been developed for scope other than the cure of neurodegenerative disorders; however, latest studies on animal models with neuronal dysfunctions report positive effects, allowing their testing in clinical trials. Drug repositioning allows clinicians to progress faster, due to the knowledge of pharmacokinetic properties, tolerability, and side effects of the drugs. The compounds reported in the following section could be repurposed for HSP treatment since they are clinically ready to be tested in term of dosage, toxicity, and side effects.

#### Rapamycin

Rapamycin (RM) (Figure 2), or sirolimus, is produced by the bacterium *Streptomyces hygroscopicus* and is an approved antibiotic and immunosuppressant drug used to prevent rejection in organ transplantation and for treatment in cardiovascular diseases and in certain types of cancer (Liu et al., 2019). RM inhibits the mammalian target of rapamycin complex 1 (mTORC1), a serine/threonine kinase that, under nutrient-rich conditions, blocks autophagy induction (Figure 1C). The accumulation of aberrant or misfolded protein aggregates due to defective autophagy is a common feature of several neurodegenerative disorders such as Alzheimer’s (AD), Parkinson’s (PD), and Huntington’s diseases (HD); spinocerebellar ataxias (SCA); and different forms of amyotrophic lateral sclerosis (ALS) (Menzies et al., 2015). Autophagy is a conserved intracellular catabolic process that delivers cytoplasmic constituents to lysosomes for degradation and recycling, through the formation of double-membrane vacuoles, termed autophagosomes (Levine and Kroemer, 2008). Autophagosome accumulation has been observed in brain samples of several neurodegenerative diseases and derives from autophagy defects that result in impaired clearance. Thus, the induction of autophagy has been proposed as a strategy to prevent or attenuate the accumulation of protein aggregates. One of the pharmacological compounds that can induce autophagy is RM.

Several studies have shown that the use of RM promotes the degradation of aggregate-prone proteins, confers neuroprotection, and improves the cognitive ability of animal models of several neurodegenerative diseases (Aso and Ferrer, 2013; Heras-Sandoval et al., 2020). Specifically, RM promotes degradation of ubiquitinated proteins and attenuates degeneration in *Drosophila* models and in dopaminergic neuronal cells from PD mouse models (Dehay et al., 2010). RM confers neuroprotection in *Drosophila* and mouse models of HD by reducing protein aggregates. Indeed, mice expressing mutant huntingtin treated with the RM analogous temsirolimus have better performance in behavioral tasks affected by neurological dysfunction (rotarod test, grip strength test, wire maneuver test, and tremors), compared with untreated mice (Ravikumar et al., 2004). Moreover, RM treatment reduces amyloid-beta levels and improves cognition in a mouse model of AD: AD mice fed with a rapamycin supplement diet for 3 months show improved learning and memory (Spilman et al., 2010). RM reduces TAR DNA-binding protein 43 (TDP-43) protein aggregates, thus having potential effects in ALS; temsirolimus injection reduces ataxin-3 inclusions and improves motor function, enhancing rotarod performance in a mouse model (Menzies et al., 2010). In addition, the administration of RM improved the pathological phenotypes of a SPG3A *Drosophila* model with muscular and neuronal cell degeneration, accumulated protein aggregates, increased oxidative stress, and early paralysis and death (Xu et al., 2017).

Neurodegenerative disorders are progressive diseases and should require a chronic administration of RM; therefore, several RM side effects should be taken into consideration. Long-term RM treatment can affect mTORC2 activity, which is associated with pro-survival pathways, impairing neuronal survival. Indeed, RM has been reported to increase amyloid toxicity, reduce long-term potentiation and synaptic plasticity, and promote brain atrophy in AD (Talboom et al., 2015) and also to exacerbate neuronal damage in a SOD1 murine model of ALS (Zhang et al., 2011). Another aspect is that the target of RM, mTOR, is involved in axonal growth, synaptic plasticity, and learning and memory; therefore, a long-lasting RM treatment and mTOR inhibition could induce cognitive defects (Garelick and Kennedy, 2011). RM has a poor solubility and stability in aqueous solutions, but its lipophilicity enables the crossing of the blood–brain barrier, which could be considered as an advantage in these cases (Zhang et al., 2011).

mTOR is also implicated in a number of cellular processes, other than autophagy, including protein synthesis, mitochondrial metabolism, and glucose and lipid metabolism, and its inhibition is not always beneficial. Therefore, RM and its analogs (sirolimus, temsirolimus, and everolimus; Figure 1) can lead to immunosuppression, altered glucose metabolism, increased risk for type 2 diabetes, lipid homeostasis alteration, and renal dysfunction (Bové et al., 2011). The efficacy and the occurrence and severity of the adverse effects of sirolimus correlate with blood concentrations; thus, its concentration in blood should be monitored during treatment (Mahalati and Kahan, 2001). Sirolimus has a low oral bioavailability (10%). It has also a long half-life that allows once-daily administration. About 98% of it is excreted in the bile in the form of metabolites and only 2% in the urine. Everolimus is a derivative of sirolimus with greater polarity, stability, and solubility (Kirchner et al., 2004). The oral bioavailability of everolimus in rats is low (16%), but higher than that of sirolimus. Everolimus is absorbed rapidly (within 30 min after drug intake) and metabolized mainly in the gut and liver by cytochrome P450 (CYP), with the same excretion of sirolimus. Everolimus has a rapid clearance and requires twice-daily administration.

Sirolimus is currently in use in one clinical trial for ALS and one for AD (clinicaltrials.gov). No trials for other neurodegenerative disorders are registered, while hundreds of trials are reported for cancer and coronary heart disease and several dozens for diabetes, anemia, angina pectoris, atherosclerosis, autoimmune diseases, and bone marrow diseases.

#### Methylene Blue

Methylene blue (MB) (Figure 2), also known as methylthioninium chloride, was originally synthesized as a dye, but later demonstrated to possess significant medical properties (Schirmer et al., 2011). MB is an oxidation-reduction agent, initially approved by the FDA for the treatment of pediatric and adult patients with acquired methemoglobinemia and successively used widely in Africa to treat malaria (Lu et al., 2018). Several evidences show that MB is effective also in the treatment of vasoplegic syndrome, hypoxia and hyperdynamic circulation in cirrhosis of the liver, severe hepatopulmonary syndrome, and ifosfamide-induced neurotoxicity; in the improvement of hypotension associated with various clinical states; and as antiseptic in urinary tract infections (Ginimuge and Jyothi, 2010). Common side effects include headache, vomiting, confusion, shortness of breath, and high blood pressure (Tucker et al., 2018). MB can be administered orally and intravenously for systemic effects. Data from animal studies showed that MB is able to pass the blood–brain barrier, with higher concentrations observed in the CNS after intravenous administration compared to oral intake (Peter et al., 2000; Walter-Sack et al., 2009). MB is a nootropic agent that inhibits acetylcholinesterase activity and at large doses also monoamine oxidase, thus increasing the levels of catecholamines and acetylcholine (Pfaffendorf et al., 1997; Ramsay et al., 2007; Delport et al., 2017). Moreover, MB has an important role in mitochondrial energy through the modulation of respiration; it accepts electrons from NADPH (becoming leukomethylene blue MBH) and transfers them to cytochrome c. Therefore, MB is used to enhance mitochondrial function, acting as an alternative electron carrier in the electron transport chain in the mitochondria (Wen et al., 2011; Gureev et al., 2019). MB also stimulates glucose metabolism, increasing glucose uptake and ATP production, providing more cellular energy for a better overall brain function including cognition, mood, and memory (Atamna et al., 2008; Rojas et al., 2012; Alda et al., 2017; Sonntag et al., 2017). Its antioxidant and neuroprotective properties led to the use of MB in a clinical trial for AD, where it was thought to reduce tau fibrillization and aggregation and to induce autophagy (Soeda et al., 2019). Today, several clinical trials are reported for the treatment of mild–moderate AD (clinicaltrials.gov). However, results in animal models are controversial. MB failed to confer protection in SOD1 and TDP43 ALS models: motor function, neuronal loss, and SOD1 aggregation were not improved (Audet et al., 2012). Moreover, MB treatment in AD mouse models after the onset of cognitive impairments has been reported to be ineffective, and only an earlier treatment preserves cognition (Hochgräfe et al., 2015). At the same time, the rescue of social behavior and of memory deficits and a reduction in beta-amyloid accumulation and in mitochondrial defects are reported with TM treatment after the onset of symptoms in AD transgenic mice (Paban et al., 2014). Regarding HSP, MB was found to target the ER stress response and protect against proteotoxicity in SPG4 animal models, including worm, fly, and zebrafish, partially improving locomotor defects, and returning ER stress marker to wild-type levels (Julien et al., 2016). Considering the increasing evidence linking HSP to ER stress and that the use of MB is already approved by FDA bring hope for a rapid translation to human therapy. At the moment, no active clinical trials are reported for neurodegenerative diseases other than AD.

#### N-Acetyl Cysteine

N-acetyl cysteine (NAC) is a precursor of cysteine, which has been used in therapy as a prodrug in the clinical treatment of paracetamol overdose for over 30 years (Ooi et al., 2018) (Figure 2). NAC is a glutathione precursor (GSH) with antioxidant and anti-inflammatory activities. The action of NAC consists in resorting the antioxidant potential by replenishing the depletion of glutathione (GSH) induced by free radical and in scavenging the reactive oxygen species. NAC is listed as an essential medicine that can be administered by inhalation or intravenously (Tardiolo et al., 2018) with a very good safety profile (Samuni et al., 2013). Gamma-glutamyl transferase-deficient mice have reduced levels of GSH in various organs and show sexual immaturity, growth retardation, and cataract development. NAC diet integration partially rescues cataracts and growth retardation of this mice model, balancing GSH level and restoring mitochondrial respiration (Lieberman et al., 1996; Will et al., 2000). NAC is also considered a medication with neuroprotective properties, with the acetylated form of cysteine being able to cross the blood–brain barrier (Yin et al., 2016). NAC can modulate key neurotransmitter systems, such as glutamate (Minarini et al., 2017), and has been used for the treatment of several neuropathies and neuropsychiatric disorders (Olive et al., 2012). NAC increases GSH levels and reduces mitochondrial damage in a cellular model of PD, decreasing dopamine-induced neuronal cell death, and is therefore a promising therapy for this disease (Monti et al., 2016). *In vivo* NAC protects brain mitochondria and counteracts age-related memory loss. Indeed, NAC treatment in aged mice increases GSH levels in the brain and the activities of the mitochondrial complex in pre-synaptic terminals, reduces oxidative damage, and increases brain synaptic and non-synaptic connections (Banaclocha, 2000, 2001; Tardiolo et al., 2018). Consistently, PD patients present a significant reduction in GSH levels in the substantia nigra, and the use of NAC in patients affects the dopaminergic system with positive clinical effects (Monti et al., 2019). NAC is being tested in some clinical trials for the treatment of PD. NAC also has been tested as a medication in AD, aiming to enhance the antioxidant response in the brain and to reduce AD toxicity (Tardiolo et al., 2018). Indeed, NAC shows a protective effect against hydroxyl radicals and oxidative damages and increases brain GSH levels in rodents (Koppal et al., 1999; Pocernich et al., 2000). Moreover, a pre-treatment with NAC of AD mice models before amyloid-β application induces an improvement in learning and memory deficits (Fu et al., 2006). Considering HSP, NAC ameliorates the phenotypes of SPG4 animal model by rescuing ER stress, locomotion, and lifespan (Julien et al., 2016).

NAC has been tested in several clinical trials for neurodegenerative disorders, alone or in combination with other compounds (Tardiolo et al., 2018). NAC displays a low oral bioavailability, and its oral administration in a PD clinical trial is reported to have no effect on brain GSH levels and to induce adverse effects, such as mild indigestion, drooling, and a mild-to-moderate increase in tremors. Conversely, intravenous administration increased GSH levels. Long-term oral administration in combination with other compounds (like folic acid and vitamins E and B12) in an AD clinical trial improves cognitive and behavioral functions.

Overall, several studies on cellular and animal models and preclinical studies suggest that NAC may be considered as a possible drug for HSP therapy.

#### Guanabenz

Guanabenz (GA, also known as Wytensin) (Figure 2) is an orally active α2-adrenergic agonist that has been initially used for the treatment of hypertension (Holmes et al., 1983). GA also showed therapeutic effects as a suppressor of prion toxicity in an animal screening, which was independent of the α2-adrenergic receptor, prolonging the lifespan (Tribouillard-Tanvier et al., 2008). GA is protective in models of neurodegeneration, reducing the ER stress response in TDP-43 *C. elegans* and *D. rerio* models of ALS (Vaccaro et al., 2013). Initially, GA was proposed to specifically interact with and inhibit GADD34 (growth arrest and DNA damage-inducible protein), a protein that induces eIF2α dephosphorylation and is involved in the integrated response to stress and protein synthesis (Balch et al., 2008). Recently, the work of Crespillo-Casado et al. (2017) showed that although GA treatment increases eIF2α phosphorylation and reduces protein synthesis activity, GA and its derivative Sephin 1 were unable to specifically inhibit the GADD34–holophosphatase complex. Moreover, another report confirmed that GA exerts its effect against eIF2α independently of GADD34 activity, affecting instead the abundance of cholesterol 25 hydroxylase (CH25H), a cholesterol hydroxylase linked to antiviral immunity (Crespillo-Casado et al., 2017). GA can promote neuronal survival in PD-related cellular models via serial up-regulation of eIF2α phosphorylation, activating ATF4 and parkin expression (Sun et al., 2018). Administration of GA improves motor performance and attenuates motor neuron loss in a SOD1 G93A mouse model of ALS, delaying the onset of the disease and extending the lifespan (Jiang et al., 2014). GA was used in a clinical trial for ALS demonstrating safety and tolerability, suggesting that it could slow down disease progression (Bella et al., 2017), and multiple sclerosis (clinicaltrial.gov). So far, no clinical trials are active for HSP, but it has been demonstrated that GA reduces ER stress and improves locomotor and cellular defects in *C. elegans*, *Drosophila*, and zebrafish SPG4 animal models (Julien et al., 2016), suggesting that GA could be another promising drug for the treatment of HSP.

### New Promising Compounds

The last compound’s section summarizes the molecules that are at the early stage of preclinical and/or clinical studies. One of the main reasons of clinical trial failure at the early stages is the arising toxicity seen in humans, compared to small/medium animal results. The second aspect engraving on clinical trial success is the difference in drug metabolism between animal models and humans (Fogel, 2018). In the following section, we discuss the potential of naringenin, which is already tested for safety and tolerability in early stage clinical trials, and salubrinal that has only been tested in preclinical models.

#### Salubrinal

Salubrinal (SAL) (Figure 2) is a selective inhibitor of eIF2α dephosphorylation that protects cells from ER stress-induced apoptosis (Boyce et al., 2005). ER stress and the activation of the UPR are common hallmarks of many neurodegenerative diseases. Increased levels of phosphorylated PERK and IRE1α have been found in the hippocampus of patients with AD (Hoozemans et al., 2005). The exposure of cultured human neurons to amyloid-β42 (Aβ42), the major component of AD amyloid plaques, induces C/EBP homologous protein (CHOP) expression and the activation of the ER-resident caspases 4/12, which are involved in ER stress-induced apoptosis, leading to neuronal death (Hitomi et al., 2004; Roussel et al., 2013). Moreover, α-synuclein accumulation on the ER of dopaminergic neurons is accompanied by UPR activation in PD models (Colla et al., 2012). SAL has been used in AD, PD, and ALS models to rescue ER stress. Treatment with SAL results in decreased toxicity of Aβ42 in primary neurons (Huang et al., 2012), while it alleviates α-synuclein accumulation and improves motor performance and lifespan of a PD model (Colla et al., 2012). Likewise, SAL reduces axon pathology and denervation in SOD1 mutant motor neurons, attenuating disease manifestation, and progression and extending the survival of ALS mouse models (Saxena et al., 2009). Moreover, SAL has been shown to protects against TDP-43 toxicity in *C. elegans* and zebrafish models of sporadic ALS (Vaccaro et al., 2013). By increasing eIF2α phosphorylation, drugs such as SAL will likely increase cellular levels of GADD34 and thereby limit the duration of their action. This could make it difficult to develop an effective dosing regimen but at the same time would reduce the possibility of overdosing, making SAL a safer drug. SAL and ER stress have been linked also to HSP. In particular, SAL treatment rescues ER stress, locomotion, and lifespan of *C. elegans*, zebrafish, and *Drosophila* SPG4 models (Vaccaro et al., 2013). Very recently, SAL provided some exciting results when tested in REEP1 null mutant mice. Wang et al. (2020) reported dramatic motor deficits in 40-week-old mutant mice, along with axonal degeneration and NMJ denervation. Similarly to the *Drosophila* model, mice lacking REEP1 exhibited a slight increase in BiP expression, a known sign of ER stress. Administration of SAL was able to restore the neuronal-associated phenotypes of REEP1 mutant, thus making SAL a promising compound for HSP therapy (Wang et al., 2020). On the one hand, SAL has no toxicity in *in vitro* experiments, but on the other hand, it has low solubility and bioavailability. Although a biotinylated derivative was obtained (Long et al., 2005), there is no evidence of improved pharmacokinetic and druggability (Fullwood and Zhou, 2012). SAL is a new compound in a preclinical study and needs to be further explored for toxicity, pharmacokinetic, and metabolism, since so far no clinical trials are registered for this compound.

#### Naringenin

Naringenin (NAR, 4’,5,7-trhydroxyflavone) (Figure 2) is one of the most important natural flavonoids derived from citrus species and tomatoes (Hernández-Aquino and Muriel, 2018; Salehi et al., 2019). The precise mechanism by which NAR acts is not well known to date, but most studies support its dual action on the regulation of antioxidants. Thanks to the chemical structure, NAR not only activates cellular antioxidant mechanisms but also has the ability to accumulate in membranes, decreasing their fluidity. In this way, the contact between the lipids and the radical species decreases and consequently there is less lipid peroxidation. In addition, NAR activates the signaling pathway mediated by nuclear activation factor 2 (Nrf2), a regulator of cellular resistance to oxidants, which has gained attention in the recent years as a target of some neurodegenerative disorders, such as AD and PD (Barone et al., 2011; Speciale et al., 2011; Murphy and Park, 2017). Nrf2 modulates the response to ER stress and autophagy and mediates the crosstalk between lipid metabolism and antioxidant defense (Jiang et al., 2015). NAR, as other flavonoids, is involved in regulating metabolism homeostasis (Fantin et al., 2019). Moreover, NAR binds to calcium channels with two-pore domains (TPC2) and to lysosomal wide conductance calcium channels (BKCa), altering the ionic homeostasis of lysosomes, increasing the concentration of Ca^2+^ and H^+^ ions (Saponara et al., 2009; Pafumi et al., 2017). The first NAR drug is sold commercially as a nutraceutical compound in the United States and is classified by the FDA agency as GRAS (generally recognized as safe). Two very recent clinical trials aimed to acquire information on the adsorption, metabolism, biodistribution, and excretion as well as on the side effects of NAR in healthy subjects. NAR and its glycosylated form, naringin, were tested in rats, dogs, and humans to compare the differences in pharmacological properties among species by analyzing metabolic and pharmacokinetic parameters. Pharmacokinetic tests showed similar parameters of NAR between rats and humans, except for a prolonged excretion process seen in humans and a better AUC (area under the curve, the variation of a drug concentration in blood plasma as a function of time) for oral administration, compared to the intravenous route (Bai et al., 2020) (chinadrugtrials.org). Moreover, oral administration of NAR from 150 to 900 mg/day, in a randomized, controlled, single ascending dose clinical trial shows no adverse effects (Rebello et al., 2020) (clinicaltrial.gov).

As previously mentioned, NAR is not well absorbed by the human gastrointestinal system, and its oral bioavailability is around 15%, due to its large adsorption in the colon (68%) (Joshi et al., 2018). Because of its chemical structure, NAR is very lipophilic and can be easily modified by environmental factors such as changes in pH, temperature, and light. For this reason, numerous formulations with drug-delivery systems have been developed and studied to improve their solubility in water, oral bioavailability, and thermal stability. Recent studies show that the formation of inclusion complexes with 2-hydroxypropyl-beta-cyclodextrin (HPβCD), an excipient approved by FDA, allows to obtain a better vehiculation of the flavonoid compound (Yang et al., 2013). These characteristics make HPβCD a promising candidate to complex with non-polar phenolic compounds such as NAR. The use and study of NAR in neurodegenerative models are relatively recent, but it has already amply demonstrated its beneficial power (Nouri et al., 2019). NAR neuroprotective role has emerged in AD *in vivo* models after ameliorating spatial learning and memory in a PI3K/AKT/GSK3β pathway-dependent manner (Khan et al., 2012). Moreover, an inclusion complex containing NAR and HPβCD tested on HSP fruit fly model of REEP1 decreases IRE1 and PERK-activated signaling, restores ER morphology and ER stress, and improves the locomotor deficit associated to the model (Napoli et al., 2019). Furthermore, NAR-HPβCD promotes peripheral nerve regeneration in mice and reduces oxidative and inflammatory damage at the site of the lesion, underlining its potential application in neuronal dysfunction (Oliveira et al., 2020); also, naringenin effectively prevents apoptosis and inhibits lipid peroxidation (Nouri et al., 2019). These last promising reports give raise to the future use of NAR in clinical studies for neurological pathologies such as spastic paraplegia conditions.

## Conclusion and Future Perspectives

HSPs, as other rare diseases, are biologically complex, and the work of the researchers in the last decade allowed to uncover an increasing number of underlying causes and the clinical course in patients; within HSP patients, many variations or subtypes result in different clinical manifestations and disease progressions. The therapeutic progress of rare diseases suffers because of low clinical trials. The genetic and biological complexity of HSP disease presents a unique barrier for scientists to design and implement a drug development program. Additionally, due to the inherently small population of patients, the recruitment for a clinical trial can be difficult. Moreover, pharmaceutical companies do not spontaneously try to develop drugs for HSP, mainly because of their limited market. Among 80–90% of clinical trials fail, because of the lack of efficacy, lack of funding, and other factors, such as failing to maintain good manufacturing protocols or to follow FDA guidance (Fogel, 2018). If we look at rare diseases, like HSPs, the situation becomes even more complicated because of the weakness of clinical trial design and the availability of biomarkers. Indeed, a biomarker is essential not only as a diagnostic tool or prognostic for the progression of the disease but also a predictive tool of the treatment/pharmacodynamic response. Despite these stumbling blocks, the drugs described in this review represent a starting point to discuss and design future clinical trials for HSPs (Augustine et al., 2013; Crow et al., 2018; Tambuyzer et al., 2020). With the identification of causative genes of HSP, a complementary line of research has been dedicated to develop HSP animal models and build a map of mechanisms and pathways involved in the onset and progression of the disease. These studies highlighted some interesting aspects of HSP mechanisms as the convergence of mutation in different genes to the same biological pathway. Herein, we have described ER homeostasis regulation and ER stress as common pathways regulated by different HSP-related genes. This report suggests that HSP patients, carrying different genetic variations involved in the same pathway, in theory, can respond to the same treatment. In light of the above, iPSC, *D. melanogaster*, *C. elegans*, and *D. rerio* have been used to screen compounds, opening the way to the development of a future therapy. iPSC-derived neuronal cell are valuable for their genetic similarity to humans for the possibility to investigate drug effects on neuronal dysfunctions such as the reduced neurite complexity, increased axonal swellings, and impaired axonal transport. Indeed, iPSCs have been established for several SPG genes and used to analyze the effect of various mitotic drugs (in SPG3a and SPG4), the liver X receptor (LXR) agonist GW3965 (SPG4), and the glycogen synthase kinase 3β (GSK-3β) inhibitor tideglusib (SPG11) with indicative relevant results (Pozner et al., 2018; Mou and Li, 2019; Rehbach et al., 2019). We believe that investing more resources on drug screening in simple HSP models can help to identify pharmacologically active substances against the disease. *In vivo* small animal models can provide interesting information concerning important parameters such as adsorption, distribution, and passage across the blood–brain barrier. Moreover, the short life cycle, the low animal costs, the ease of disease model development, the possibility of high-throughput and high content screening, and the use of simple screening assays make these small animal models a suitable tool for drug screening (Strange, 2016).

Of note, the preclinical models should be oriented toward already approved FDA drugs with a potential for a faster application in clinical trials. This implies a preliminary screening of compounds that satisfies the basic features of a safety and tolerable drug. Considering the pharmacokinetics and pharmacodynamics of the newly identified compound in HSP preclinical models, some of them can be considered in future clinical trials. A second emerging aspect of HSP to be considered is the overlapping pathways of HSP neurodegeneration mechanisms with other type of diseases, such as AD, ALS, and PD. Taking into consideration these information, we can speculate that the compounds already in clinical trials for ALS or AD, such as MB, RM, NAC, and GA, could be also considered for HSP patients. Finally, a combinational therapy, taking advantage of the different effects of these compounds on the cellular component, could be an interesting strategy to develop in order to fight different cellular defects at the same time; using compounds that simultaneously target ER stress and mitochondrial energy could be a turning point in HSP therapy. Moreover, the use of compounds that target lysosomal defects should be considered as an alternative strategy to address ER stress, and their efficacy should be analyzed in ER-related HSP models. In particular, two classes of molecules seem to be more promising for these HSP forms: compounds that reduce lipids synthesis and accumulation in lysosomes, such as miglustat, eliglustat tartrate, venglustat, and lucerastat (Poswar et al., 2019; Felis et al., 2020; Parenti et al., 2021), and compounds that target TFEB, a master regulator of lysosome biogenesis and function (Settembre et al., 2011), such as resveratrol (Shao et al., 2021). Among these drugs, only miglustat, a glucosylceramide synthase inhibitor approved for Niemann-Pick disease type C (NPCD) therapy that reduces cholesterol and ganglioside accumulation in lysosomes (Pineda et al., 2018), has been tested in an HSP form and has been shown to improve the motor phenotype in a Spg11 zebrafish model (Boutry et al., 2018). Further analyses are needed for this class of compounds.

Finally, along with the implementation of drug screening and multidrug combination efficacy, other aspects should be implemented, such as the selection of appropriate efficacy endpoint(s) (quantifiable biomarkers such as 27-hydroxycholesterol level in serum for SPG5), to have a direct readout of clinical benefit.

## Author Contributions

SG, CV, and GO contributed in the drafting and refining of the manuscript. GO and MM contributed in the critical reading of the manuscript. All of the authors have read and approved the manuscript.

## Conflict of Interest

The authors declare that the research was conducted in the absence of any commercial or financial relationships that could be construed as a potential conflict of interest.
